# Regulation of *Leishmania (L.) amazonensis* Protein Expression by Host T Cell Dependent Responses: Differential Expression of Oligopeptidase B, Tryparedoxin Peroxidase and HSP70 Isoforms in Amastigotes Isolated from BALB/c and BALB/c Nude Mice

**DOI:** 10.1371/journal.pntd.0003411

**Published:** 2015-02-18

**Authors:** Priscila Camillo Teixeira, Leonardo Garcia Velasquez, Ana Paula Lepique, Eloiza de Rezende, José Matheus Camargo Bonatto, Marcello Andre Barcinski, Edecio Cunha-Neto, Beatriz Simonsen Stolf

**Affiliations:** 1 Heart Institute (InCor), University of São Paulo School of Medicine, São Paulo, São Paulo, Brazil; 2 Department of Parasitology, Institute of Biomedical Sciences, University of São Paulo, São Paulo, Brazil; 3 Department of Immunology, Institute of Biomedical Sciences, University of São Paulo, São Paulo, Brazil; 4 Department of Biochemistry, Institute of Chemistry, University of São Paulo, São Paulo, Brazil; 5 Oswaldo Cruz Institute-FioCruz, Rio de Janeiro, Brazil; University of Notre Dame, UNITED STATES

## Abstract

Leishmaniasis is an important disease that affects 12 million people in 88 countries, with 2 million new cases every year. *Leishmania amazonensis* is an important agent in Brazil, leading to clinical forms varying from localized (LCL) to diffuse cutaneous leishmaniasis (DCL). One interesting issue rarely analyzed is how host immune response affects *Leishmania* phenotype and virulence. Aiming to study the effect of host immune system on *Leishmania* proteins we compared proteomes of amastigotes isolated from BALB/c and BALB/c nude mice. The athymic nude mice may resemble patients with diffuse cutaneous leishmaniasis, considered T-cell hyposensitive or anergic to *Leishmania*´s antigens. This work is the first to compare modifications in amastigotes’ proteomes driven by host immune response. Among the 44 differentially expressed spots, there were proteins related to oxidative/nitrosative stress and proteases. Some correspond to known *Leishmania* virulence factors such as OPB and tryparedoxin peroxidase. Specific isoforms of these two proteins were increased in parasites from nude mice, suggesting that T cells probably restrain their posttranslational modifications in BALB/c mice. On the other hand, an isoform of HSP70 was increased in amastigotes from BALB/c mice. We believe our study may allow identification of potential virulence factors and ways of regulating their expression.

## Author Summary

Leishmaniasis is an important disease that affects 12 million people in 88 countries. *Leishmania amazonensis* is an important agent of leishmaniasis in Brazil, leading mainly to localized (LCL) and sometimes to diffuse cutaneous leishmaniasis (DCL), depending on the host immune response to infection. We believe that host immune response affects not only the clinical form and survival of *Leishmania*, but also the phenotype and virulence of the parasite. To analyze the effects of the host on *Leishmania* phenotype, we compared protein expression (proteome) of parasites isolated from wild type mice and from mice lacking T cells. We identified some protein isoforms differentially expressed, which may further be studied as potential virulence factors.

## Introduction

Leishmaniasis is an important disease that affects 12 million people in 88 different countries in Europe, Africa, Asia and America, and 2 million new cases are reported every year (WHO 2004; [1,2]. There are different forms of tegumentary and visceral leishmaniasis, that depend on the *Leishmania* species and on the genetic/immunologic status of the host, all transmitted to man by the bite of naturally infected species of phlebotomine sand flies [3].

In Brazil, *Leishmania braziliensis* and *Leishmania amazonensis* are considered the main pathogenic species causing human tegumentary leishmaniasis [4]. The human *L. amazonensis* infection may lead to different clinical forms, varying from the localized cutaneous leishmaniasis (LCL), with moderate cellular hypersensitivity, to the diffuse cutaneous leishmaniasis (DCL), frequently associated to anergy to parasite’s antigens [4,5].

The murine model has been commonly used to analyze several aspects of *Leishmania* infection such as the virulence of different parasite species [3,6] and how different mouse strains respond to the same *Leishmania* species [7,8,9,10]. The infection of mice by *Leishmania major* has been the most commonly used model, and allowed the definition of resistant and susceptible lineages such as C57BL/6 and BALB/c, which mount Th1 and Th2 responses, respectively [11,12]. In infections by *L. amazonensis* the dichotomy of susceptible and resistant mice is not evident. In fact, most lineages are susceptible to this *Leishmania* species [3,13] and develop a mixed Th1-Th2 response to the parasite, producing IL-4 and IFNγ [6,11]. However, some differences can be observed in the progression and size of lesions according to the strain [7,8]. The low and mixed Th1/ Th2 responses seen in *L. amazonensis*-infected mice are similar to those observed in human infections [4], validating the biological relevance of these mouse models to study the human disease [14]. The response to *Leishmania* infection in athymic nude mice, however, has not been thoroughly analyzed. Nude mice of C57BL/6 background have been shown not to develop lesions when infected by *L. amazonensis*, and had an expected reduced influx of T cells and monocytes in the site of infection [15]. No similar analysis has been performed to date in BALB/c nude mice. These observations suggest that immunopathology of *Leshmania* infections should be better characterized.

One interesting issue difficult to study in human infections and rarely analyzed in mouse model is how the host immune response affects *Leishmania* phenotype and virulence. One example of modulation already described is phosphatidylserine (PS) exposure in *L. amazonensis* amastigotes. The display of PS in the external membrane is an apoptotic feature that leads to parasite intracellular survival due to inhibition of macrophage inflammatory response [16,17]. It has been shown, that the host immune response modulates PS exposure by *L. amazonensis* amastigotes so that parasites derived from the more susceptible BALB/c mice display more PS than parasites derived from less susceptible C57BL/6 mice [17]. Accordingly, PS exposure was positively correlated with clinical parameters of the human infection (number of lesions and time of disease) and with characteristics of the experimental infection such as macrophage infection and anti-inflammatory cytokine induction [18].

Other amastigote molecules besides PS are certainly modulated by the host immune response. Since *Leishmania* and other trypanosomatids lack a conventional network of transcription factors and most genes are constitutively transcribed [19,20], most changes in *Leishmania* phenotypes are better studied in terms of proteins [21].

The analysis of cell proteomics is an efficient method to compare protein profiles. Most studies of *Leishmania* proteomes compared abundance or post-translational modifications (specially phosphorylation) of proteins in amastigotes and promastigotes of the same *Leishmania* species [20,22,23,24,25,26], parasites sensitive and resistant to drugs [27,28,29,30], and proteins from different *Leishmania* species [31,32]. Some works also analyzed immunogenic proteins [22,33,34] and secreted proteins [34,35,36,37]. Due to the difficulty to obtain robust amounts of virulent amastigotes from infected animals for *in vitro* analysis, most works have used axenic amastigotes for proteomics analysis [23,26,35]. However, comparison of proteomes from lesion derived amastigotes and axenic amastigotes have shown important differences among them [38,39].

Aiming to evaluate the effect of the host immune system on protein expression in *Leishmania*, we analyzed proteomics of mouse lesion-derived amastigotes employing the protein separation by two-dimensional electrophoresis with fluorescent labeling (DIGE) and protein identification by mass spectrometry (MALDI-ToF/ToF) approach. We compared the proteomes of amastigotes isolated from BALB/c and BALB/c nude mice. The immune system of nude mice, in which T lymphocytes are nearly absent [40], may shed some light on the immune system of patients bearing the diffuse cutaneous leishmaniasis, who are anergic individuals [4].

## Materials and Methods

### *Leishmania amazonensis* promastigotes

Promastigotes of *Leishmania amazonensis* LV79 strain (MPRO/BR/72/M 1841-LV-79) were cultured at 24°C in Warren medium with 10% FCS. Parasites were subcultured every 7 days for 2x10^6^/mL.

### Mice infection

Four to 8-week-old female BALB/c and BALB/c nude female mice were bred under specific- pathogen free conditions at the Isogenic Mouse Facility of the Parasitology Department, University of São Paulo, Brazil. Mice were infected in one of the hind footpads with 2 x 10^6^ stationary-phage promastigotes of *L.amazonensis* strain LV79 (MPRO/BR/72/M 1841-LV-79). Footpad thickness was measured weekly using a caliper.

### Ethics statement

All animals were used according to the Brazilian College of Animal Experimentation (CONEP) guidelines, and the protocols were approved by the Institutional Animal Care and Use Committee (CEUA) of the University of São Paulo (protocol number 001/2009).

### Amastigote purification and lysis

Thirteen weeks after infection the animals were sacrificed and the lesions were removed under sterile conditions. Amastigote isolation was performed as described by Wanderley et al., 2006. Briefly, lesions were minced and homogenized in 5ml PBS using a tissue grinder (Thomas Scientific). After centrifugation at 50 x *g* for 10 min at 4°C, the supernatant was recovered and centrifuged at 1450 x *g* for 17 min at 4°C. Supernatant was then removed and the pellet was washed three times with PBS followed by centrifugations at 1450 x *g* for 17 min at 4°C. After 3h incubation under rotation at room temperature to liberate endocytic membranes (8), amastigotes were further centrifuged, resuspended in 2mL of erythrocyte lysis buffer (155mM NH4Cl, 10mM KHCO3, 1mM EDTA, pH7,4) and incubated for 2min in ice.

This method has been previously validated for isolation of amastigotes free from macrophage contaminants (Balanco et al., 2001).

For lysis parasites were washed twice in PBS, resupended at 10^9^ cells/300μl in PBS+Proteoblock 1x (Fermentas) and lysed by 8 cycles of freeze thaw in liquid nitrogen-42°C. Soluble proteins were obtained after centrifugation at 12.000xg for 3 minutes, concentrated for ~5mg/ml using Microcon 5K (Millipore) and quantified by Bradford (Biorad).

### Two-dimensional electrophoresis

50 μg of extracts (adjusted to pH 8–9) of amastigotes from BALB/c or BALB/c nude were labeled by “minimal labeling” with 1ul (400pmol) of N-hydroxysuccinimidyl-ester-derivates of the cyanine dyes Cy3 or Cy5 (GE Healthcare) for 30min on ice. The reaction was quenched with 1ul of 10 mM lysine for 10 min on ice. 50 μg of a pool of all samples was similarly labeled with Cy2 (GE Healthcare) as an internal standard. The three differently labeled extracts were pooled and incubated with Immobiline DryStrips pIs 4–7 (linear, 13cm, GE Healthcare) in the presence of 7M urea, 2M tiourea, 15mM TrisHCl, 2% CHAPS, 0,5% IPG 4–7 and 3μl of DeStreak reagent for 16h. This procedure was performed for the preparation of analytical gels. Furthermore, for subsequent identification of proteins, a preparative gel was also performed, applying a sample containing 500μg of protein from the Pool (450 ug of unlabeled protein and 50 ug labeled protein with Cy2) to the IPG strip by rehydration. Isoelectric focusing was performed at: 300V for 4h, 500V until 0,5kVh, 1000V until 0,8kVh, 8000V until 18,7kVh, 300V for 2h, at a maximum current of 50μA/strip. Focused IPG strips were incubated for 15 min in equilibration solution (75 mM Tris-HCl, pH 8.8, 6 M urea, 30% glycerol, 2% SDS) with 10mg/mL DTT and then the proteins were alkylated for further 15 min in equilibration solution containing 25mg/mL iodoacetamide. Strips were transferred to 12% SDS-PAGE gel and eletrophoresis was performed at 30mA for 30min and 60mA for the remaining time. Directly after the second dimension, the fluorescent gels were scanned. The preparative gel was fixed in a solution containing 40% methanol and 10% acetic acid, and then stained with Deep Purple stainer according to the manufacturer’s instructions (GE Healthcare). The gels were kept in a solution containing 1% citric acid.

For 2D Western blots, 120μg of extracts were used in each 7cm pI 4–7 Strip (GE healthcare). Strips were rehydrated in DeStreak solution containing one of the samples for 16h. Focusing was performed as recommended for these strips and equilibration and alkylation were done as described above. Second dimension was done in 12% SDS-PAGE gel and Western blot was performed as described below.

### Acquisition of images from two-dimensional gels

The gels containing samples labeled with fluorophores were scanned using "Typhoon 9410 Variable Mode Imager” (GE Healthcare), with the following parameters: Cy2, 488nm excitation and 520nm-BP 40nm emission filters; Cy3, 532nm excitation and 580nm-BP 40nm emission filters; Cy5, 633nm excitation and 670nm-BP 40nm emission filters. For Deep Purple-stained gels, 532nm excitation and 610nm-BP 30nm emission filters were used. The gels were scanned with resolution 100 micra and the sensitivity ranged from 450 to 550 PTM.

### Analysis of differential protein expression

The analysis of differential protein expression using the DIGE technique was performed using the program "DeCyder Differential Analysis (GE Healthcare). The volume of each spot was normalized in relation to the total volume of spots selected for that labeling (sample), and the gels were normalized together using the image of the pool of samples labeled with Cy2. Statistical analysis, through the One Way Anova and the Student’s test, were performed to compare protein expression in different samples. Spots were considered to be differentially expressed if p<0.05. We considered a protein to be differentially expressed if one or more of the associated spots were differentially expressed.

### Protein identification

Selected spots were collected automatically using Ettan Workstation (GE Healthcare), transferred to 96 well plates and kept at -20°C until shipping. Samples were analyzed at Institut Pasteur of Montevideo. Peptide mass fingerprinting was carried out by in-gel trypsin treatment (Sequencing-grade Promega) overnight at 37°C. Peptides were extracted from the gels using 60% acetonitrile in 0.2% TFA, concentrated by vacuum drying and desalted using C18 reverse phase micro- columns (OMIX Pippete tips, Varian). Peptide elution from micro-column was performed directly into the mass spectrometer sample plate with 3 μl of matrix solution α-cyano-4-hydroxycinnamic acid in 60% aqueous acetonitrile containing 0.2% TFA). Mass spectra of digestion mixtures were acquired in a 4800 MALDI-TOF/TOF instrument (Applied Biosystems) in reflector mode and were externally calibrated using a mixture of peptide standards (Applied Biosystems). Collision-induced dissociation MS/MS experiments of selected peptides were performed. Proteins were identified by NCBInr database searching with peptide m/z values using the MASCOT program and using the following search parameters: monoisotopic mass tolerance, 0.05 Da; fragment mass tolerance, 0.25 Da; methionine oxidation, as possible modifications; and one missed tryptic cleavage allowed.

### Sample preparation for flow cytometry analysis

BALB/c and BALB/c nude female mice were infected with *Leishmania amazonensis* as described above. Thirteen weeks after infection, mice were euthanized and spleens, popliteal lymph nodes, and paws were harvested for single cell suspension preparations and flow cytometry analysis. Uninfected animals with similar ages were used as controls. All tissues were mechanically dissociated in MTH (Mouse Tonic Hanks: 1x HBSS, 15 mM HEPES pH 7.4, 0.5 U/ml DNase I, 5% fetal bovine serum). Splenocytes were treated with hypotonic buffer for red cell lysis. After washing and counting, 10^6^ cells from each tissue were aliquoted, incubated with Fc Block (BD Biosciences, San Jose, CA) for 10 min in ¼ of the final staining volume. Antibodies were added to blocked cell suspensions and incubated for 20 min, on ice. After washing in MTH, cells were fixed in 4% formaldehyde in PBS. Antibodies used for cell labeling were: FITC-conjugated anti-CD11c, Alexa 647-conjugated anti-CD8, PE-conjugated anti-CD4, biotin-conjugated anti-CD19, APC-conjugated anti-CD3, PE-conjugated anti-Gr1 (BD Biosciences, San Jose, CA), FITC-conjugated anti-CD11b (R&D Systems, Minneapolis, MN), PECy5.5-conjugated anti-F4/80, PECy5.5-conjugated anti-rat IgG (eBiosciences, San Diego, CA). We also used Alexa 647-conjugated streptavidin (LifeTechnologies, former Molecular Probes, Carlsbad, CA). Cell staining was performed to identify mainly myeloid populations (CD11b, Gr1, F4/80, CD3) and mainly lymphoid populations (CD4, CD8, CD19, CD11c). Cells were analyzed in a FACSCalibur flow cytometer (BD Biosciences, San Jose, CA), where at least 30,000 events per sample were acquired. Data was analyzed with the FlowJo software (TreeStar, Ashland, OR).

### Western blot

10ug of soluble amastigote proteins were separated in 12% acrylamide gels and transferred to nitrocellulose membranes (GE healthcare) using a semidry system (GE healthcare). Membranes were incubated in PBS with 5% milk and 0.1% Tween 20 for one hour and with primary antibodies (anti-SHP70, anti-OPB or anti-TXNPx) in PBS with 2.5% milk and 0.1% Tween 20 for two hours. Three washing steps with PBS 1x 0.1% Tween 20 for 10min were performed and followed by incubation with secondary antibodies diluted in PBS with 2.5% milk and 0.1% Tween for one hour. Membranes were washed twice in PBS 1x 0.1% Tween 20 and once in PBS, 10min each. After incubation with ECL Prime Western Blotting Detection Reagent (GE healthcare) for five minutes, membranes were exposed to X-Ray films. Images (in TIF files) were analyzed using ImageJ software and the results were normalized to GAPDH band intensities.

### Immunohistochemistry in footpad lesions

Footpads from mice infected for 13 weeks were fixed in paraformaldehyde 4% at 4°C for 18h and after washing and dehydration in ethanol were infiltrated with xylene and paraffin. 4μm paraffin sections were used for immunohistochemistry. After deparaffinization and blocking with 5% BSA in PBS for 30 minutes, endogenous peroxidase was blocked in 0,1% sodium azide, 3% H_2_O_2_ in methanol for 30 minutes. After incubation with the primary antibodies in PBS 2% (w/v) BSA for 18h at 4°C, the samples were washed in PBS and incubated with secondary peroxidase-conjugated antibodies for two hours, followed by washings in PBS. They were next incubated with DAB (DAKO, Glostrup, Denmark), counterstained with hematoxylin, dehydrated and diafanized, and mounted with Permout (Sigma).

### Statistical analysis

Intensities of DIGE spots of amastigotes from BALB/c and nude mice were compared using t test of DeCyder BVA Software. Western blot ratios of samples from BALB/c and nude mice were also compared using t test.

Statistical analyses of FACs data was done using ANOVA followed by Tukey´s multiple comparison test for all comparisons except for lymphoid and dendritic cells in footpads, where t test was employed. * = p<0.05.

## Results

### Lesion progression in BALB/c and BALB/c nude mice infected with *L. amazonensis*

**Fig 1:**
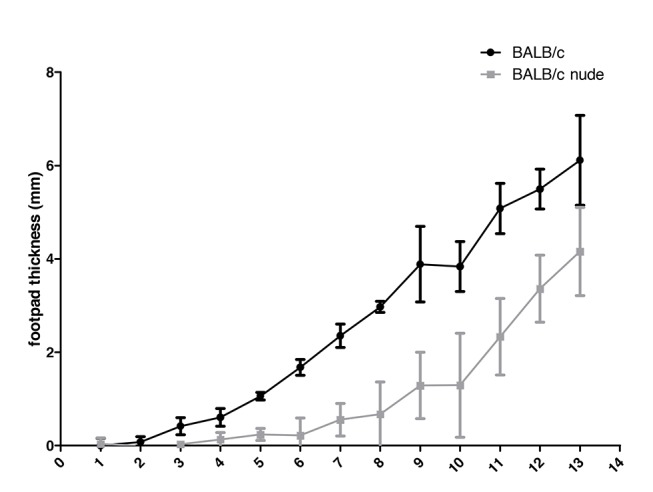
Footpad lesion sizes (difference between infected and non-infected footpads) in mm of BALB/c (n = 6) and BALB/c nude (n = 5) mice infected with *L. amazonensis*, measured weekly for 13 weeks.

Mice were infected in the left footpads and lesion size was estimated by measuring left footpad thickness weekly. As shown in Fig. 1, footpad thickness increased earlier in BALB/c than in BALB/c nude mice and was larger in the former than in the latter during the thirteen weeks of monitoring.

The difference between BALB/c and BALB/c nude footpad thickness does not seem to reflect parasite numbers and the establishment of infection after 13 weeks. In fact, quantification of parasite loads in the footpads showed slightly higher numbers of amastigotes in BALB/c nude lesions (mean values of 1.51x10^8^ and 4.94 x 10^8^ parasites for BALB/c and nude mice, respectively), although the difference was not statistically significant.

### Cell populations in lesions, spleen and draining lymph nodes of BALB/c and BALB/c nude infected mice

**Fig 2:**
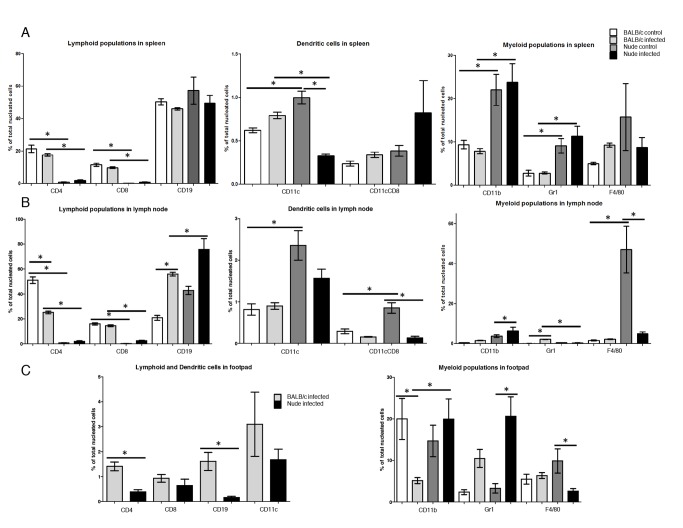
Comparison of lymphoid, myeloid and dendritic cell populations in spleens (A), popliteal lymph nodes (B) and footpads (C) of infected and non infected BALB/c and BALB/c nude mice by flow cytometry. Results of one experiment with 3 animals of each for control condition and four of each for infection. Statistical analyses by ANOVA followed by Tukey´s multiple comparison test for all comparisons except for lymphoid and dendritic cells in footpads (Fig. 2C left), where t test was employed. * = p<0.05

To better characterize the difference between the wild type and the athymic mice, we compared immune cell populations in spleens, draining lymph nodes (popliteal) and footpads in infected and non-infected (control) mice. Using flow cytometry, we quantified the percentages of the following leukocyte populations: T lymphocytes as CD3^+^CD4^+^ or CD3^+^CD8^+^ cells, monocytes as CD11b^+^ (Mac-1) cells, macrophages as CD11b^+^F4/80^+^, polymorphonuclear cells (PMNs), mainly neutrophils, as CD11b^+^Gr1^+^ cells, DCs as CD11b^+^CD11c^+^ or CD11b^+^CD11c^+^CD8^+^, and finally B lymphocytes as CD19^+^ cells. The frequency of the above populations in spleen, lymph nodes and footpads in control and infected BALB/c and nude mice are shown in Fig. 2.

The frequencies of CD4 and CD8 T cells were higher in spleens and lymph nodes of control BALB/c than in nude mice, as expected (Fig. 2A and B). CD4 corresponds to 21% and 0,8% of the cells in spleens of BALB/c and nude, respectively, and CD8 to 12 and 0.2%. In lymph nodes of BALB/c and nude CD4 corresponds to 50% and 0.8% of the cells, respectively, and CD8 to 16 and 0.2%. The number of cells in uninfected footpads was very low and insufficient for the labeling of all markers. We therefore analyzed only myeloid cells in these tissues (Fig. 2C), so that we could compare to nude mice, which display mainly myeloid cells.

Fig. 2A shows that infection did not significantly change the frequency of CD4 and CD8 T cells, as well as B lymphocytes, in the spleen. Monocytes (CD11b^+^) and polymorphonuclear cells (Gr1^+^) were more abundant in nude mice spleens before and after infection (CD11b around 22% and Gr1 around 10% in nude, versus 8 and 3% in BALB/c), possibly due to the low numbers of T cells. Their proportions did not change after infection. Macrophages had similar abundances in BALB/c and nude mice spleens, before and after infection.

In popliteal lymph nodes from BALB/c infected mice, there was a significant decrease in the frequency of CD4 T cells (from 50 to 25%, Fig. 2B). Infection significantly increased B cells in BALB/c lymph nodes (from 20 to 55%), but infected nude showed higher proportions of these cells (75%) than wild type mice. This result is expected, since we are working with percentages of total cells and nude mice virtually lack T lymphocytes. Dendritic cells CD8^+^ and CD8^-^ were more frequent in control nude lymph nodes (0.8 and 2.3%, respectively, versus 0.3 and 0.8% in BALB/c), and DC CD8+ population decreased significantly (to 0.1%) in these mice after infection. As observed for spleens, monocytes were more common in infected nude than in BALB/c lymph nodes (6.4 versus 1.7%), but differently from spleen, polymorphonuclear cells increased in BALB/c mice after infection and became significantly more abundant than in infected nude mice (2.3 versus 0.4%). Macrophages were more frequent in uninfected nude than in BALB/c mice (47 versus 1.6%), but decreased significantly (to 4.9%) in the athymic lineage after infection.

In infected footpads the wild type and athymic mice had similar proportions of CD8^+^ T cells and dendritic cells (Fig. 2C). B cells and CD4^+^ T cells, on the other hand, were less abundant in BALB/c nude lesions (0.2 and 0.4% versus 1.6 and 1.4% in BALB/c, respectively). Footpads showed recruitment of some cell populations after infection, although a more complete analysis was hampered by the low number of cells recovered from control tissue, as already mentioned. BALB/c had a decrease in the proportion of monocytes after infection, while nude mice had increased frequency of polymorphonuclear cells and a decrease in macrophages in the lesions (Fig. 2C).

### Comparison of lesion-derived amastigotes proteomes by DIGE

**Fig 3:**
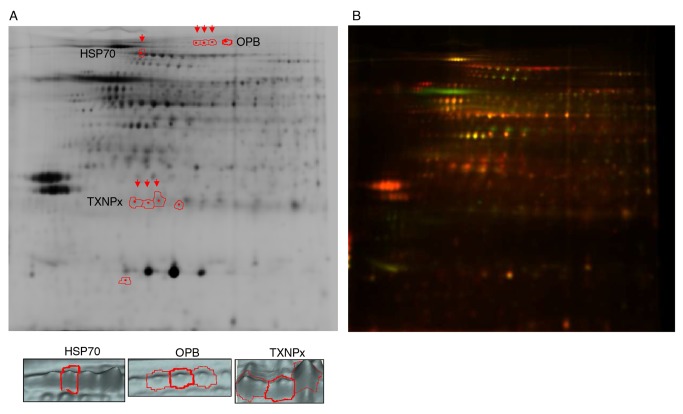
Representative 2D gel images. A. Negative (black and white) image of a pool of all samples labeled with Cy2, highlighting the spots corresponding to differentially expressed HSP70, OPB and TXNPx isoforms and the 3D images of the spots marked with arrows. B. Representative image of one experiment with amastigote samples from BALB/c and BALB/c nude labeled with Cy5 (red) and Cy3 (green), respectively

Soluble proteins from amastigotes isolated from BALB/c and BALB/c nude mice lesions were analyzed by DIGE. Fig. 3 shows a representative image of one experiment showing labeling of a pool of all samples (3A) and differential labeling of samples isolated from the two mouse strains (3B). In Fig. 3A spots corresponding to isoforms differentially expressed in the two samples are highlighted and represented in 3D images.

In all gels labeling of Cy3 and Cy5 was normalized with the Cy2 labeled pool of all samples (shown in Fig. 3A). Spots detected by the software were manually adjusted to exclude artifacts. The reproducibility and technical accuracy was verified by comparison of labeling of the pool of all samples with Cy3 and Cy5. Considering a cut-off of two fold for differential expression, 99,20% of the 1944 spots included in the analysis were similarly labeled by the two dyes. This cut-off was therefore employed for the experimental comparisons.

The DeCyder DIA module detected and matched 2100–2300 spots in each gel, that after manual validation were reduced to about 1700 spots. The DeCyder BVA module was then employed to compare the differentially expressed spots out from a total of 1178 spots that were matched considering the three experiments. According to our data set, amastigotes isolated from the two mouse strains share over 96% of the protein expression profile (p<0.05). The differences that can be attributed to the presence of T cell dependent responses are linked to only 3.4% (40 spots) of the proteins, which have decreased (18 spots) or increased (22 spots) abundance in BALB/c nude derived amastigotes. These spots were selected for identification by MS analysis. This analysis was performed with spots collected from preparative gels containing a pool of the protein samples from the three experiments. Due to the low abundance of most of these proteins and incompleteness of *Leishmania* protein databases, only 21 spots yielded protein identifications. These spots are listed in Table 1.

**Table 1:** Identity of the differentially expressed spots.

MW gel (kDa)	pI gel	T-test	Ratio (N/B)	Hit (gi)	Protein Identification	MW (kDa)
84	6	0.077	1.44	146335222	oligopeptidase B from *Leishmania amazonensis*	83.4
84	5.7	0.093	1.33	146335222	oligopeptidase B from *Leishmania amazonensis*	83.4
84	5.8	0.027	1.36	146335222	oligopeptidase B from *Leishmania amazonensis*	83.4
84	5.8	0.004	1.53	146335222	oligopeptidase B from *Leishmania amazonensis*	83
75	5.2	0.048	-1.81	729766	Heat shock 70 kDa protein from *Leishmania amazonensis*	71
71	5.3	0.033	-2.04	123665	Heat shock protein 83 from *Leishmania amazonensis*	81
62	5.5	0.02	2.27	146086185	beta tubulin from *Leishmania infantum*	49
60	4.9	0.38	1.59	13569565	beta-tubulin from *Leishmania mexicana*	49
				606648	alpha tubulin from *Leishmania donovani*	49
60	5.8	0.048	1.43	104745490	trypanothione reductase from *Leishmania amazonensis*	51
				606648	alpha tubulin from *Leishmania donovani*	49
58	5.7	0.013	1.74	322489395	alanine aminotransferase from *Leishmania mexicana*	54.9
58	5.8	0.007	2.29	322490429	metallo-peptidase Clan MG, Family M24 from *Leishmania mexicana*	42.4
53	4.7	0.059	1.54	7689258	glucose-regulated protein 94 from *Leishmania infantum*	86
33	5.4	0.083	-1.81	322488901	hypothetical protein from *Leishmania mexicana*	25.9
30	5.9	0.036	-1.66	2654167	activated protein kinase C receptor homolog LACK from *Leishmania donovani*	30
23	5.4	0.018	2.23	322491865	peroxidoxin from *Leishmania mexicana*	25
				145411494	cytoplasmic tryparedoxin peroxidase from *Leishmania donovani*	22
22	5.3	0.044	1.79	145411494	cytoplasmic tryparedoxin peroxidase from *Leishmania donovani*	22
22	5.2	0.054	2.53	145411494	cytoplasmic tryparedoxin peroxidase from *Leishmania donovani*	22
22	5.6	0.13	1.4	62183806	mitochondrial peroxiredoxin from *Leishmania donovani*	25
				145411494	cytoplasmic tryparedoxin peroxidase from *Leishmania donovani*	22
20	5	0.064	2.4	157868848	hypothetical protein from *Leishmania major*	44
12	5.1	0.077	-1.62	157781821	cytosolic tryparedoxin from *Leishmania donovani*	16
21	5.4	0.007	1.81	154339774	small GTP-binding protein Rab1 from *Leishmania braziliensis*	22
				322489864	tryparedoxin peroxidase from *Leishmania mexicana*	22

Spot number (ordered based on molecular weights), experimental molecular weight (MW gel) and isoeletric point (pI gel), p value obtained after T-test (T-test), ratio of intensity between amastigotes from nude/BALB/c (N/B), gene identification (gi) of the Hit, protein identification and its respective MW.

Among the differentially expressed spots comparing amastigotes from BALB/c and BALB/c nude mice we observed many proteins associated with oxidative/nitrosative stress (trypanothione reductase, peroxidoxin, tryparedoxin peroxidase, tryparedoxin, heat shock proteins) or proteins with protease/peptidase activity (oligopeptidase B, metallo-peptidase). Among them there are some known virulence factors of *Leishmania* such as oligopeptidase B [41,42] and tryparedoxin peroxidase [39,43,44]. Four isoforms of oligopeptidase B and four isoforms of tryparedoxin peroxidase were differentially expressed (Table 1 and Fig. 3A); all of them overexpressed in nude mice derived parasites. Isoforms of proteins such as HSPs 70 and 83 were less abundant in nude derived parasites (Table 1 and Fig. 3A) and are known to have chaperone function upon increased temperature and oxidative stress in differentiating and proliferating amastigotes [23]. Different isoforms of alpha and beta-tubulin were more abundant in nude-derived amastigotes. Interestingly, these cytosketetal proteins were also shown to be more expressed in antimonial resistant *L. braziliensis* and *L. infantum* [30].

**Fig 4:**
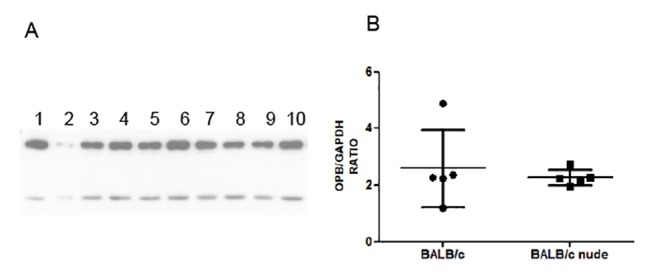
Western blot for OPB expression in 5 pairs of lesion derived amastigotes from BALB/c and BALB/c nude mice. A. Western blot image showing OPB (upper band) and GAPDH (lower band) in soluble extracts from BALB/c derived amastigotes (1–5) and BALB/c nude derived amastigotes (6–10). B. Expression of OPB relative to GAPDH in the 10 samples. Statistical analysis by t-test. * = p<0.05

**Fig 5:**
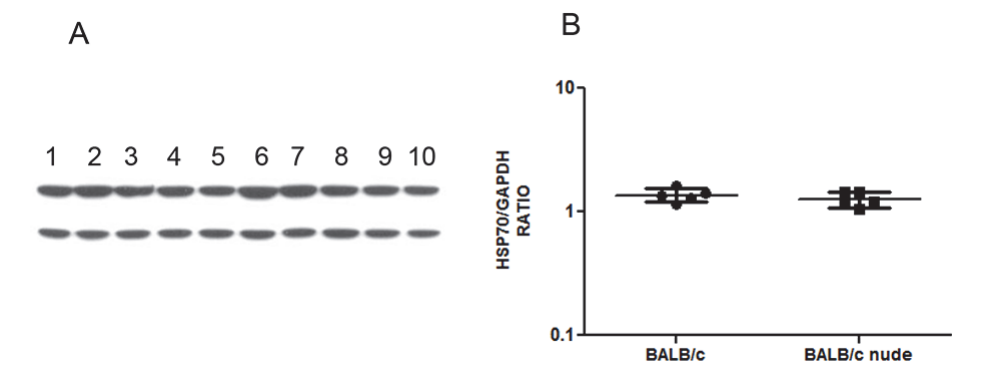
Western blot for HSP70 expression in 5 pairs of lesion derived amastigotes from BALB/c and BALB/c nude mice. A. Western blot image showing HSP70 (upper band) and GAPDH (lower band) in soluble extracts from BALB/c derived amastigotes (1–5) and BALB/c nude derived amastigotes (6–10). B. Expression of HSP70 relative to GAPDH in the 10 samples Statistical analysis by t-test. * = p<0.05

**Fig 6:**
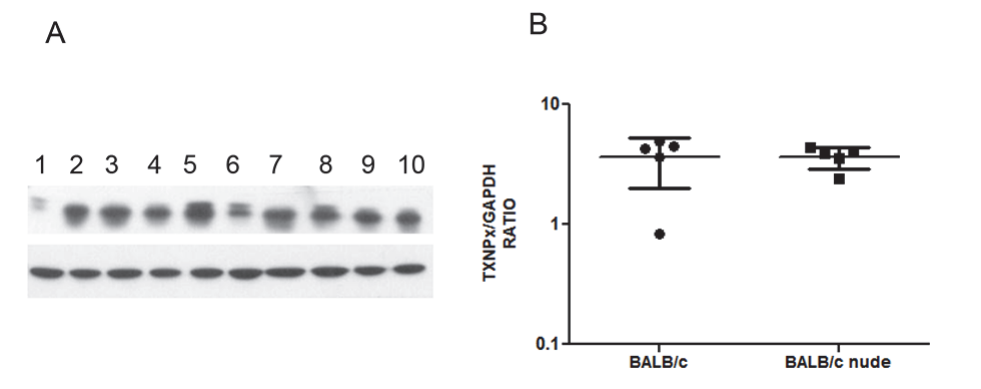
Western blot for tryparedoxin peroxidase (TXNPx) expression in 5 pairs of lesion derived amastigotes from BALB/c and BALB/c nude mice. A. Western blot image showing tryparedoxin peroxidase (upper band) and GAPDH (lower band) in soluble extracts from BALB/c derived amastigotes (1–5) and BALB/c nude derived amastigotes (6–10). B. Expression of tryparedoxin peroxidase relative to GAPDH in the 10 samples. Statistical analysis by t-test. * = p<0.05

We next evaluated the expression of OPB, TXNPx and HSP70 in five pairs of BALB/c and BALB/c nude derived amastigotes by Western blot. Figs. 4–6 show Western blot images and densitometric quantifications for OPB, HSP70 and tryparedoxin peroxidase, respectively.

As can be observed, amastigotes from the two mouse strains show similar expression of OPB, TXNPx and HSP70. OPB and TXNPx had four isoforms overexpressed in nude-derived parasites and HSP70 one isoform decreased in nude-derived amastigotes. The lack of correspondence between 2D-DIGE and conventional Western blots highlights the importance of analyzing isoforms of a protein individually.

**Fig 7:**
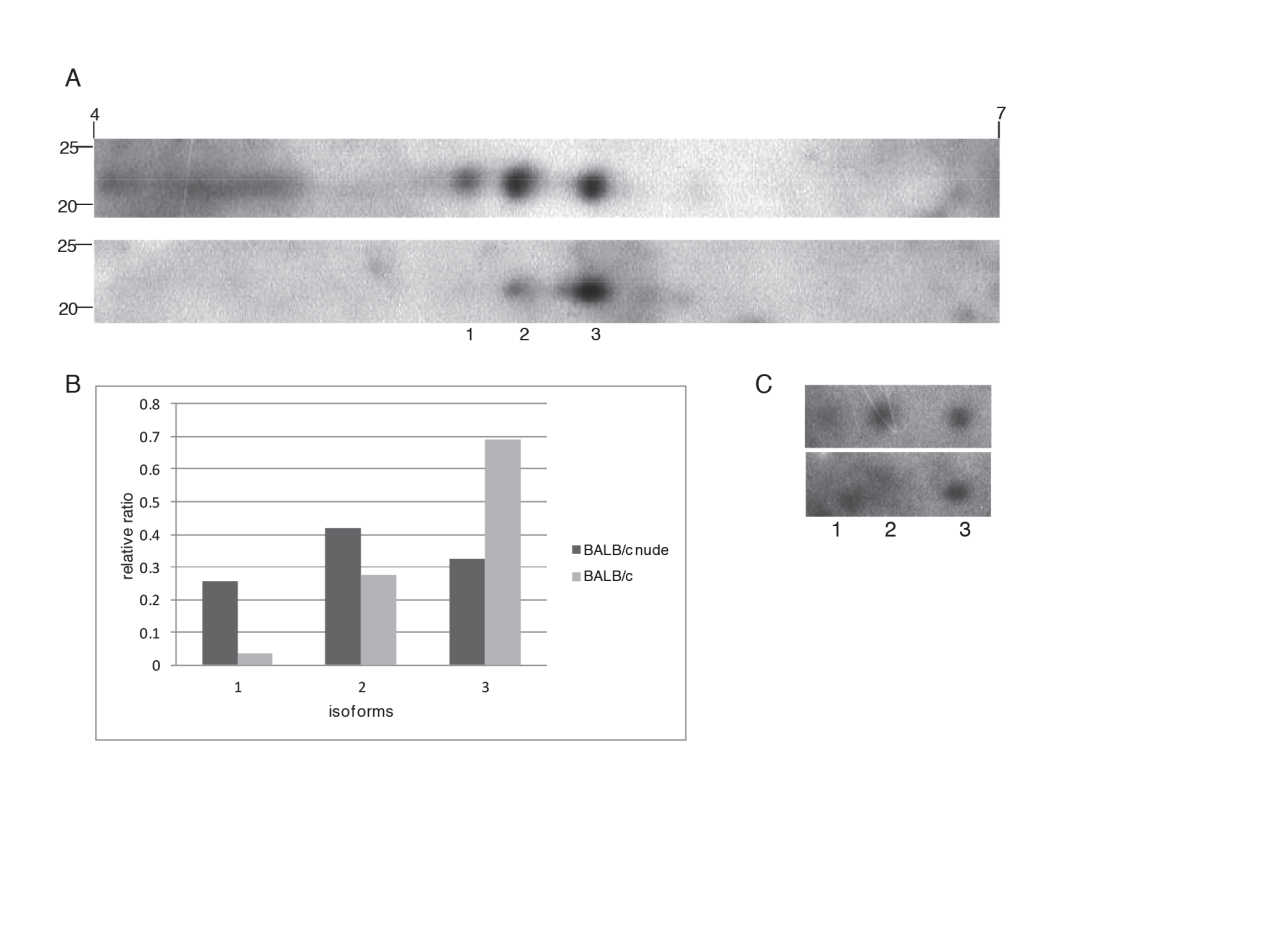
2D Western blot for TXNPx in amastigotes from BALB/c and BALB/c nude mice. A. Western blot image showing three isoforms (1, 2, 3) in soluble extracts from BALB/c nude (upper image) and BALB/c (lower image) derived amastigotes. B. Relative abundance of each isoform after normalization (considering the sum of three isoforms similar in the two samples). C. Western blot image showing labeling of the isoforms with anti-phospho S, T, Y pooled antibodies in the membranes shown in A.

**Fig 8:**
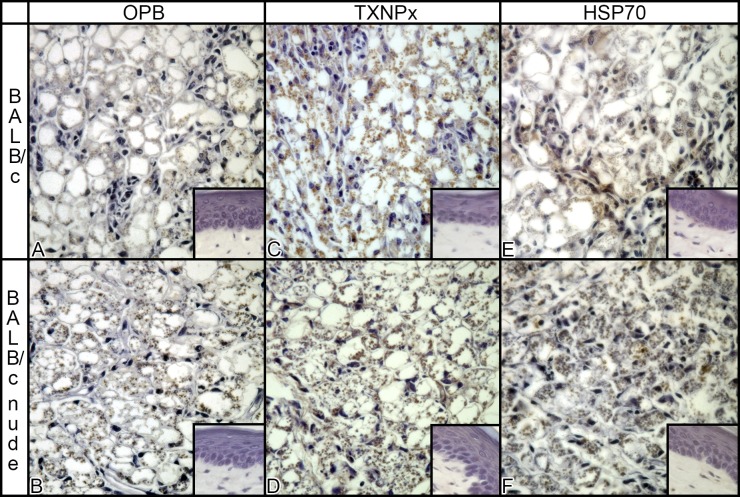
Immunohistochemistry for OPB, TXNPx and HSP70 in infected and non infected (small pictures) footpads from BALB/c and BALB/c nude mice.

To confirm the differential abundance of isoforms of TXNPx in parasites isolated from BALB/c and nude lesions, we performed 2D Western blots for this protein in a pair of samples. Three isoforms were identified in both samples, with pIs of 5.22 5.39 5.65 (Fig. 7A). Since the total amount of TXNPx was shown to be similar in the two types of extract by conventional Western blot (Fig. 6), we considered the sum of the three spots similar in the two samples and calculated the relative abundance of each isoform. The data shown in Fig. 7B indicate higher abundance of isoforms with pIs 5.2 and 5.39 in amastigotes isolated from BALB/c nude, confirming DIGE findings (Table 1). The same membranes were incubated with anti-phospho S, T, Y antibodies, and the observed labeling of the three isoforms (Fig. 7C) indicate that there are phosphorylated in at least one of these residues. Besides analyzing OPB, TXNPx and HSP70 expression in parasite extracts from lesions (Figs. 4, 5, 6)), we analyzed the abundance of the three proteins in sections of lesions from infected BALB/c and BALB/c nude mice. As shown in Fig. 8, all proteins are visible in amastigotes inside macrophages of both mice, and no labeling is observed in non-infected footpads. These results indicate that the proteins are specifically expressed by the parasites in both hosts.

## Discussion

Interaction between pathogens and their hosts derives from long term co-existence. Intracelular pathogens such as *Leishmania* respond to an aggressive microenvironment in the host creating evasion mechanisms that guarantee their survival. To further explore the adaptation between *Leishmania* and its host, we have studied the protein expression profile of parasites harvested from footpad infections in immunocompetent BALB/c mice, and immunodeficient BALB/c nude mice. Our results indicate that there may be a correlation between the cellular immune response of the host and specific protein isoforms of the parasite.

Footpad thickness increased in BALB/c and in BALB/c infected mice, but with a delay in the athymic animals. This difference is probably due to the observed and already described low number of T cells in the nude mice [15], leading to a compromised IFNγ production, inflammation in the site of infection and parasite clearance. Lesion progression patterns similar to our wild type mice were observed in BALB/c infected with other *L. amazonensis* strains [11,45,46].

Comparison of cellular compositions in lesions and lymphoid organs of mice indicate that *L. amazonensis* amastigotes in infected BALB/c and BALB/c nude footpads face different cells and consequently a distinct inflammatory milieu. As expected, lymphoid organs and lesions of nude mice had very low percentages of T lymphocytes. The lack of T cell responses impairs effector responses against the parasite, leading to uncontrolled *Leishmania* growth in the footpads of nude mice. On the other hand, lack of T cell responses may also lead to uncontrolled innate inflammation, as observed in HIV patients with very low CD4 T cell levels [47,48]. In our experimental model, we observed changes in the frequency of leukocytes in peripheral lymphoid organs of naïve and infected mice, mainly in the lesion draining lymph nodes. Of particular interest, we observed a significant decrease in the frequency of CD4 T cells and increase of B cells and Gr1 cells in BALB/c mice. In nude mice, there was a significant decreased in myeloid populations CD11cCD8 dendritic cells and macrophages, probably due to cell death induced by the known high parasite loads in these cells [49,50]. Interestingly, in footpads we observed a decrease of CD11b cells of BALB/c infected mice and macrophages in nude mice. However, there was a robust increase in the granulocyte population in infected nude mice, which could be indicative of an inflammatory response against the parasite.

The different pattern of cells and cytokines in the lesions of BALB/c and BALB/c nude mice resulted in 3.4% differential expression of soluble proteins in amastigotes. About half of the proteins that were significantly differentially expressed in the three experiments were identified, and many of them were related to oxidative/nitrosative stress or had protease/peptidase activity. Specific isoforms of trypanothione reductase, peroxidoxin, cytoplasmic tryparedoxin peroxidase (different isoforms), oligopeptidase B (different isoforms), alanine aminotransferase, metallo-peptidase, and small GTP-binding protein Rab1 were increased in BALB/c nude derived amastigotes. On the other hand, isoforms of heat shock 70 kDa protein, heat shock 83 kDa protein and a smaller form of cytosolic tryparedoxin were decreased in BALB/c nude derived parasites.

Infection of macrophages leads to production of cytotoxic oxidants, and *Leishmania* must be able to detoxify these agents to survive and proliferate inside the cell. Similarly to the other trypanosomatids but differently from other eukaryotes and prokaryotes, *Leishmania* has a trypanothione redox system instead of the more ubiquitous glutathione/glutathione reductase (GR) system [51]. Trypanothione participates in the detoxification of hydroperoxides, metals and drugs and in the synthesis of DNA precursors. The molecule is used as a donor of electrons and reduces the hydroperoxides generated by macrophages during infection [52]. Tryparedoxin peroxidase (TXNPx) catalyzes the detoxification reaction, and trypanothione reductase regenerates the reduced dithiol state of trypanothione necessary for all these reactions [51]. Recent data showed that trypanothione reductase, tryparedoxin peroxidase and peroxidoxin were 1.2 to 2 fold in *Leishmania donovani* promastigotes under oxidative and/or nitrosative stress *in vitro* [43,53]. TXNPx also participates in oxidative resistance in *L. donovani* [43], in *L. infantum* [54] and in *L. amazonensis* [55], and increases infection of *L. donovani* and survival in the presence of antimonials [43]. Accordingly, splenic amastigotes of *L. donovani* express higher levels of the enzyme than axenic amastigotes and are more resistant to H2O2 [39], and a more virulent strain of *L. donovani* expressed more of two specific cTXNPx isoforms than a less virulent strain [56]. High levels of cTXNPx were observed in *L. donovani* isolates [57], *L. braziliensis* and *L.chagasi* lines [30] unresponsive to antimony, in *L. amazonensis* resistant to arsenite [55] and in metastatic *L. guyanensis* [58]. In our study, four TXNPx isoforms were overexpressed in nude derived parasites. It is important to analyze whether the differentially expressed isoforms are functional and whether the enzyme activity is modulated in amastigotes by the host immune system.

Isoforms of Oligopeptidase B (OPB) were also identified as overexpressed in nude-derived amastigotes. This enzyme is a serine peptidase restricted to bacteria, plants and trypanosomatids that hydrolyses peptides of up to 30 amino acids after basic residues, especially arginine [59]. OPB has been considered a virulence factor in trypanosomatids, including *Leishmania* [41,42,60]. In fact, promastigotes of *L. major* deficient in OPB did not differentiate normally to metacyclic form, showed reduced infection and survival in macrophages *in vitro* [41] and a significant delay in lesion development in vivo [42]. OPB has already been described in *L.(L.) major* [41,42,61], *L. braziliensis* [62], *L. donovani* [26] and *L. amazonensis* [63]. No previous study has described isoforms of OPB, and we have no clues about their impact on the enzyme function.

Differently from OPB and the trypanothione related proteins mentioned above, some proteins were less expressed in parasites from athymic nude mice, such as heat shock 70 and heat shock 83 kDa proteins. HSP70 and HSP83 are evolutionarily conserved, constitutively transcribed and regulated at post-transcriptional level [64]. HSPs protect cells against different types of stimuli that can cause cell damage. HSP70 assists in protein translation and translocation across membranes, avoids aggregation of damaged proteins and reactivate denatured proteins. HSP70 may protect from toxic environmental conditions by cooperating with other stress-induced proteins to prevent heat-induced denaturation prior to protein aggregation and by suppressing programmed cell death that would be triggered by the activation of specific kinases [65]. The control of HSP70 activity in *Leishmania* is regulated not only by the protein abundance, but also by phosphorylation at specific residues [23]. When expressed in response to stress encountered in mammalian host, HSPs are likely to confer protection to the parasite and to play a crucial role in their survival [66]. In fact, HSP83 increased in response to heat shock and in the initial hours of promastigote- amastigote differentiation in *L. infantum* [64] and was shown to control differentiation in *L. donovani* [67], and HSP70 and HSP83 levels are higher in *L. donovani* amastigotes compared to promastigotes [24]. Both HSP 70 and 83 were overexpressed in *L. infantum* and *L. braziliensis* resistant to antimonials [30]. HSP70 has been shown to be increased in *L. infantum* under a heat shock or sub lethal oxidative stress, and the overexpression of this HSP conferred increased resistance to H_2_O_2_ [65]. Similarly, virulent promastigotes of *L. donovani* exposed to NO showed appreciable increase in relative synthesis of HSPs 83, 70 and 65 [66], and a more virulent strain of *L. donovani* had higher abundances of three isoforms of HSP70 [56]. Different combinations of oxidative and nitrosative stresses increased in 1.3 to 1.8 fold the expression of 13 heat shock proteins (HSPs) in *L. donovani*, including HSP70 and 83 [53]. In agreement with the induction of HSPs in stress conditions, our data shows that one isoform of HSP70 was less abundant in nude derived parasites. It remains to be shown whether the increase in this isoform confers survival advantages to amastigotes, or if this isoform is a less active form of HSP70. The sequencing of *L. amazonensis* genome has recently shown that this species has a higher number of genes containing HSP70 domain compared to other *Leishmania* [68]. This information reinforces the importance of studying this gene in the context of *L.amazonensis* infection and host cell interaction.

The increased expression of specific trypanothione reductase, cytoplasmic tryparedoxin peroxidase and oligopeptidase B isoforms in amastigotes from nude mice suggest that T cells or T cell-derived mediators and cellular interactions are associated with post-translational modifications of these proteins in BALB/c infected footpads. Another possibility is that the lack of T cell suppression in nude mice could lead to higher production of reactive oxygen and/or nitrogen species by macrophages, that could stimulate the modification of trypanothione-associated enzymes, as already described [53]. Analysis of infected footpads indicated higher levels of iNOS mRNA in BALB/s than nude mice (Velasquez et al., manuscript in preparation). Comparison of ROS should also be done to better define the stress conditions faced by the parasite in the two mice. Interestingly, we found that isoforms of HSPs 70 and 83 had the opposite regulation, being under expressed in nude-derived parasites. As discussed by others [65], we believe that *Leishmania* have redundant mechanisms for surviving oxidative stress. Accordingly, we postulate that different factors must stimulate the production of TXNPx and OPB isoforms in nude-derived amastigotes (or repress them in BALB/c-derived parasites) and induce the modifications of HSP in BALB/c parasites.

Conventional Western blot experiments did not show differences in the expression of OPB, TXNPx and HSP70 between BALB/c and BALB/c nude-derived amastigotes. Since we showed that these three proteins had several isoforms, the differences noted for specific isoforms were probably compensated when the sum of all isoforms was analyzed in conventional Western blots. These results suggest that post-translational modifications (PTMs) but not total levels of the three proteins are modulated by the presence of T cells and cytokines in mice lesions. The most well characterized PTMs in *Leishmania* are phosphorylations, while less information is available on pathways and roles of methylations, acetylations and glycosylations [26]. The isoforms of these three proteins differentially expressed in the amastigotes may correspond to one or more of these PTMs, which may lead to a more or a less active form of the protein that may affect parasite survival and lesion progression. 2D Western blot for TXNPx confirmed the higher abundance of two of the four isoforms identified in DIGE experiments in amastigotes from nude mice. The other two were not labeled by the antibody, possibly due to lower abundances. These three isoforms were also recognized by anti-phospho threonine, tyrosine and serine antibodies, suggesting that phosphorylation is the PTM process that generated the different isoforms of the enzime.Different isoforms of HSP70 and TXNPx were also differentially regulated in *L. donovani* strains with different virulences, but the significance of these findings and the PTM involved is still unknown [56].

Since infected nude mice could partially reproduce the immune response of a patient with diffuse cutaneous leishmaniasis, it is important to analyze the activities and roles of the protein isoforms over expressed in this mouse strain. It is also important to search for the specific stimuli that drive the post-translational modifications of HSP70, OPB and TXNPx. We are currently attempting to generate *L. amazonensis* clones over expressing TXNPx or OPB to evaluate the contribution of these proteins to infection by this important parasite species.
